# Brachyury and Related Tbx Proteins Interact with the Mixl1 Homeodomain Protein and Negatively Regulate Mixl1 Transcriptional Activity

**DOI:** 10.1371/journal.pone.0028394

**Published:** 2011-12-02

**Authors:** Lloyd A. Pereira, Michael S. Wong, Sue Mei Lim, Alexandra Sides, Edouard G. Stanley, Andrew G. Elefanty

**Affiliations:** 1 Differentiation and Transcription Laboratory, Peter MacCallum Cancer Centre and the Pathology Department, The University of Melbourne, Melbourne, Victoria, Australia; 2 Monash Immunology and Stem Cell Laboratories, Monash University, Clayton, Victoria, Australia; University of Medicine and Dentistry of New Jersey, United States of America

## Abstract

Mixl1 is a homeodomain transcription factor required for mesoderm and endoderm patterning during mammalian embryogenesis. Despite its crucial function in development, co-factors that modulate the activity of Mixl1 remain poorly defined. Here we report that Mixl1 interacts physically and functionally with the T-box protein Brachyury and related members of the T-box family of transcription factors. Transcriptional and protein analyses demonstrated overlapping expression of Mixl1 and Brachyury during embryonic stem cell differentiation. *In vitro* protein interaction studies showed that the Mixl1 with Brachyury associated via their DNA-binding domains and gel shift assays revealed that the Brachyury T-box domain bound to Mixl1-DNA complexes. Furthermore, luciferase reporter experiments indicated that association of Mixl1 with Brachyury and related T-box factors inhibited the transactivating potential of Mixl1 on the *Gsc* and *Pdgfr*α promoters. Our results indicate that the activity of Mixl1 can be modulated by protein-protein interactions and that T-box factors can function as negative regulators of Mixl1 activity.

## Introduction

The *Mix/Bix* family of transcription factors are defined by a highly conserved 60 amino acid DNA binding motif, the homeodomain (HD), that binds preferentially to an inverted iteration of the canonical homeobox binding site, ATTA, separated by three nucleotides [1]. Mix/Bix proteins function predominantly as transcriptional activators; a function mediated through their conserved carboxy-terminal polar/acidic region [2]–[7].

Members of the Mix/Bix family play key roles in vertebrate mesoderm and endoderm formation in response to the TGFβ ligands, BMP4 and *nodal*/*activin* [2], [8]–[14]. In the mouse, the single *Mix* gene homologue, *Mix-like 1* (*Mixl1*), is expressed in the primitive streak and emerging mesendoderm [4], [12], [15]. The requirement for *Mixl1* for normal germ layer formation is demonstrated by the observation that *Mixl1*-null mouse embryos display an enlarged primitive streak and die at embryonic day 8.5, exhibiting numerous defects in mesoderm and endoderm patterning [12], [16]. Consistent with this, during embryonic stem cell differentiation in vitro, *Mixl1* and its human ortholog (*MIXL1*) mark mesendodermal precursors [13], [14], [17] and enforced expression of *Mixl1* perturbs the normal allocation of cells to the mesodermal and endodermal compartments [6], [18].

Like *Mixl1*, the *Tbx* transcription factor genes are also involved in the regulation of germ layer induction and patterning [19]. The defining feature of this family is the presence of a highly conserved DNA binding domain called the T-box. *Brachyury* (*T*), the founding member of the T-box (Tbx) family, is a transcriptional activator and is expressed throughout the nascent mesoderm, tailbud and notochord [20]–[23]. Like *Mixl1*-null embryos, *Brachyury* deficient embryos lack tail and trunk structures and die shortly after gastrulation, displaying several mesodermal abnormalities including an enlarged primitive streak [24]. Analysis of *Brachyury*-null embryos also suggests that *Brachyury* is essential for the proper specification of mesodermal cell identity and for their correct movement through the primitive streak [25]–[28].

As noted above, loss- and gain-of-function studies in the mouse suggest *Mixl1* and *Brachyury* are involved in common processes during early development. In *Xenopus*, *Mix.1* and the Brachyury homologue, *Xbra*, repress each other's expression [3], [29]. Furthermore, RNAi-mediated knockdown of *Mixl1* expression in mouse ESCs results in an enhancement of *Brachyury* expression whilst *Mixl1* over-expression suppresses *Brachyury* expression [30]. These results are consistent with the increased and prolonged expression of *Brachyury* in the expanded primitive streak of *Mixl1*-null embryos [16].

Additional members of the T-box family have also been implicated in modulating the function of *Mixl1*. *Eomesodermin (Eomes)* plays a key role in the formation early mesoderm and trophoectoderm [31], [32] as well as in the development of endodermal lineages [33], [34]. Notably, *Mixl1* expression is lost in *Eomes* null-embryos and *Eomes* and *Mixl1* also act as a negative regulators of *Brachyury* expression [30].

Despite the importance of Mix/Bix proteins during development, our understanding of the molecular mechanisms underlying their relationship with other transcription factors remains poor. In this study we show that Mixl1, Brachyury and related Tbx factors are co-expressed during embryonic stem cell differentiation. We provide evidence that Mixl1 physically interacts with Brachyury and other members of the Tbx family. Luciferase reporter experiments indicate that this association inhibits the ability of Mixl1 to activate the *Gsc* and *Pdgfr*α promoters, suggesting a functional co-operativity between Mixl1 and Tbx factors during early mammalian development.

## Results

### Co-expression of Mixl1 and Brachyury in differentiating mouse ESCs

**Figure 1:**
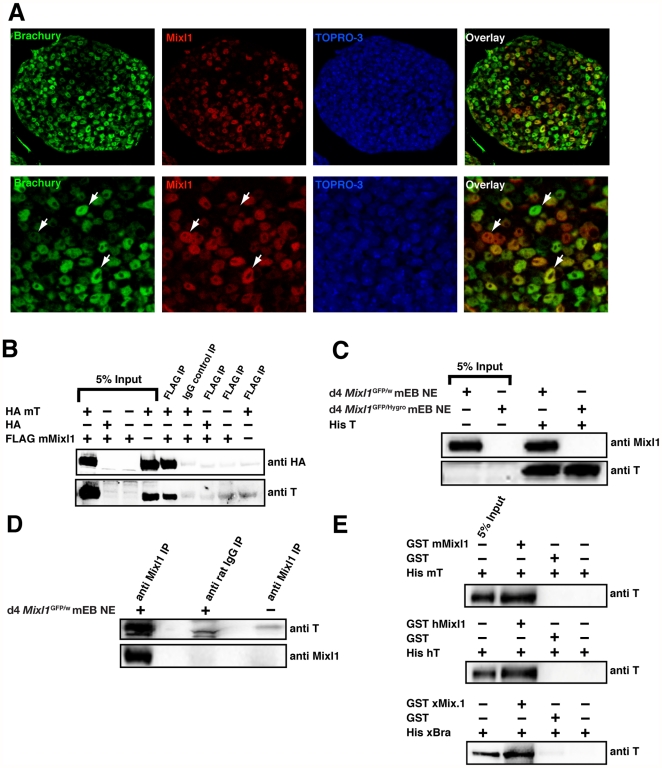
The Mixl1 homeodomain protein is co-expressed and interacts with Brachyury. (A) Immunofluorescence analysis of day 4 differentiated W9.5 mouse ESCs showing expression of Brachyury (green) and Mixl1 (red). Arrowheads indicate cells in which Mixl1 (red in overlay panel) or Brachyury (green in overlay panel) predominated, or in which expression of the two proteins was approximately equal (orange in overlay panel). Nuclei were visualized with TOPRO (Blue). Original magnification: ×50 upper row and ×100 lower row. (B) Mixl1 and Brachyury (T) associate. 293T cells were co-transfected with FLAG mMixl1 and HA mT expression plasmids and Mixl1 immunoprecipitated (IP) from whole cell lysates with anti-FLAG antibody or IgG control antibody followed by Western blot analysis with anti-HA or anti-T N19 antibodies. (C) Western blot analysis of nuclear proteins from day 4 differentiated *Mixl1* ^GFP/w^ and *Mixl1* ^GFP/Hygro^ ESCs isolated with TALON resin pre-coated with recombinant His-tagged T protein. Bound proteins were visualized using anti-Mixl1 6G2 or anti-T N19 antibodies. (D) Nuclear proteins from day 4 differentiated *Mixl1* ^GFP/w^ (**+**) or *Mixl1* ^GFP/Hygro^ (**−**) ESCs were subjected to IP with anti-Mixl1 6G2 antibody or rat isotype control antibody. Immunoprecipitates were analysed by Western blot using anti-Mixl1 2D10 or anti-Brachyury N19 antibodies. (E) The Mixl1-Brachyury interaction is conserved across vertebrate species. Recombinant His-mBrachyury (mT), hBrachyury (hT) or Xbra (xBra) were incubated with recombinant GST, GST-mMixl1, hMixl1 or xMix.1 proteins pre-adsorbed to glutathione resin. The bound fractions were analysed by Western blot with anti-T N19 antibody.

We have previously demonstrated that the transient expression of *Mixl1* RNA during the in vitro differentiation of mouse (m) embryonic stem cells (ESCs) closely mirrored the kinetics of expression of the *Brachyury* transcription factor [13], [35]. It was unclear whether this overlap reflected the presence of mesendodermal precursors that co-expressed both genes or the temporal coincidence of two distinct populations. Immunofluorescence analysis of Mixl1 and Brachyury expression in day (d) 4 ESC-derived embryoid bodies (EBs) revealed a high frequency of Mixl1^+^ and Brachyury^+^ cells, with expression of both proteins restricted to the nucleus (Figure 1A). Many cells co-expressed Mixl1 and Brachyury, with some exhibiting more intense Mixl1 staining whilst others displayed higher levels of Brachyury (Figure 1A). This pattern of staining was not observed in d9 EBs that no longer expressed Mixl1 or Brachyury (Figure S1A). These data indicated the presence of a Mixl1^+^Brachyury^+^ population of cells transiently during ESC differentiation.

### Physical association between Mixl1 and Brachyury

We wondered whether the pattern of expression observed in Figure 1A reflected a functional relationship between Mixl1 and Brachyury during development that required their physical interaction. Consistent with this hypothesis, immunoprecipitation of cell lysates prepared from 293T cells expressing epitope-tagged forms of Mixl1 and Brachyury revealed that Mixl1 interacted with Brachyury (Figure 1B). The selectivity of this interaction was argued by the finding that Mixl1 formed homodimers or heterodimers with Goosecoid (Gsc) (Figure S2), consistent with prior work [1], [2], [36], but could not heterodimerize with the POU-homeodomain protein Oct4 (Figure S2).

We next determined whether Brachyury formed a stable interaction with endogenous Mixl1. We performed poly Histidine (His) pull-down experiments with nuclear protein extracts derived from d4 EBs (Figure 1C). Recombinant His-Brachyury immobilised on Talon-affinity metal resin was able to interact with endogenous Mixl1 (Figure 1C). In contrast, no Mixl1 signal was detectable using nuclear extracts prepared from Mixl1-null d4 EBs (Figure 1C). Furthermore, in d4 EB nuclear extracts, endogenous Brachyury could be detected in an immunoprecipitated complex of endogenous Mixl1 (Figure 1D). A direct interaction between Mixl1 and Brachyury was demonstrated using purified recombinant Glutathione-S-Transferase (GST) Mixl1 and His-Brachyury fusion proteins (Figure 1E). We found that GST-Mixl1 could pull-down His-Brachyury using mouse, human and *Xenopus* forms of each protein, highlighting that this interaction was conserved across vertebrate evolution (Figure 1E).

### Mapping Mixl1 and Brachyury interacting domains

**Figure 2:**
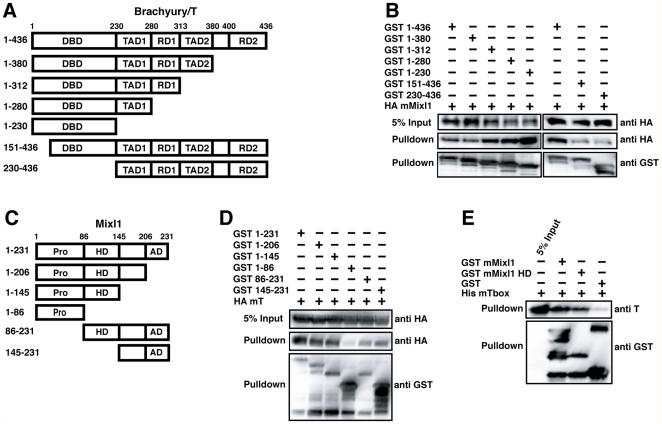
Mapping the interacting domains of Mixl1 and Brachyury. (A) Schematic of Brachyury (T) and its truncated mutants. Amino acid residues present in each protein are shown. DNA binding domain (DBD), Transactivation domain (TAD), Repression domain (RD). (B) The amino terminal DBD (T-box) domain of T and a carboxy terminal region can independently interact with Mixl1. 293T cells were transfected with HA mMixl1 and GST-T or its truncated mutants as indicated. GST-T proteins were isolated from whole cell extracts using glutathione resin and the bound fraction analysed by Western blot with an anti-HA antibody. Expression of each protein was confirmed with anti-GST and anti-HA antibodies. (C) Schematic of Mixl1 and its truncated mutants. Amino acid residues present in each protein are indicated. Proline rich domain (Pro), Homeodomain (HD), Activation domain (AD). (D) The Mixl1 HD and amino terminal flanking sequences are required for its interaction with T. 293T cells were transfected with HA mT and GST-Mixl1 or its truncated mutants as indicated. GST-Mixl1 proteins were isolated from whole cell extracts using glutathione resin and the bound fractions were analysed by Western blot with an anti-HA antibody. Expression of each protein was confirmed with anti-GST and anti-HA antibodies. (E) The Brachyury T-box domain and Mixl1 HD associate. Recombinant His mT-box was incubated with recombinant GST, GST mMixl1 or GST mMixl1 HD proteins pre-adsorbed to the glutathione resin. The bound fractions were analysed by Western blot with anti-T N19 and anti-GST antibodies.

To delineate the Mixl1 binding regions in Brachyury, a deletion analysis of Brachyury was performed. Prior mapping of key domains within the Brachyury protein identified an N-terminal (aa 1–229) DNA binding domain (DBD) (T-box), central (aa 230–380) trans-activation (TAD) and repression domains (RD) and a C-terminal (aa 400–436) second repression domain [23] (Figure 2A). Whole cell protein lysates were prepared from 293T cells expressing GST-tagged deletion mutants of Brachyury and full-length HA-tagged Mixl1 and used in GST-pulldown experiments. This analysis showed that full-length Mixl1 could interact with Brachyury deletion mutants retaining the first 230 amino acids, suggesting the amino terminal DBD is sufficient for interaction with Mixl1 (Figure 2B). Mutant proteins with amino terminal truncations (aa 151–436, aa 230–436) also bound Mixl1, albeit less robustly. Thus, Brachyury appears to have a second region outside the T-box DBD that is capable of independently interacting with Mixl1 (Figure 2B).

We mapped the domains in Mixl1 (Figure 2C) that interacted with Brachyury using similar GST-pulldown assays. We found that Brachyury bound strongly to deletion mutants that retained the homeodomain, more weakly to amino terminal truncation mutants (aa 86–231 and aa 145–231), but poorly to an isolated amino terminal Proline rich domain (aa 1–86) (Figure 2D). Thus, Mixl1 also appears to have a second region within its carboxy terminus that is capable of independently interacting with Brachyury (Figure 2D).

The in vitro binding data suggested an interaction between the DNA binding domains of Mixl1 and Brachyury. Indeed, pulldown experiments confirmed that recombinant GST-Mixl1 HD (aa 86–145) could associate with the T-box domain (aa 1–230) of Brachyury (Figure 2E). Together these results indicate that multiple regions within Mixl1 and Brachyury, including their DNA binding domains, are able to mediate association between these two proteins.

### Mixl1 DNA binding activity is not required for its association with Brachyury

Mixl1 binds its consensus DNA binding site as a cooperative dimer [1], [2], [5], [6]. To examine whether Mixl1 DNA binding activity was required for its interaction with Brachyury, we assayed a series of Mixl1 DNA binding HD mutants (P126I, V132A, I113R and R143I) [37] for their ability to associate with Brachyury [2], [5], [11].

**Figure 3:**
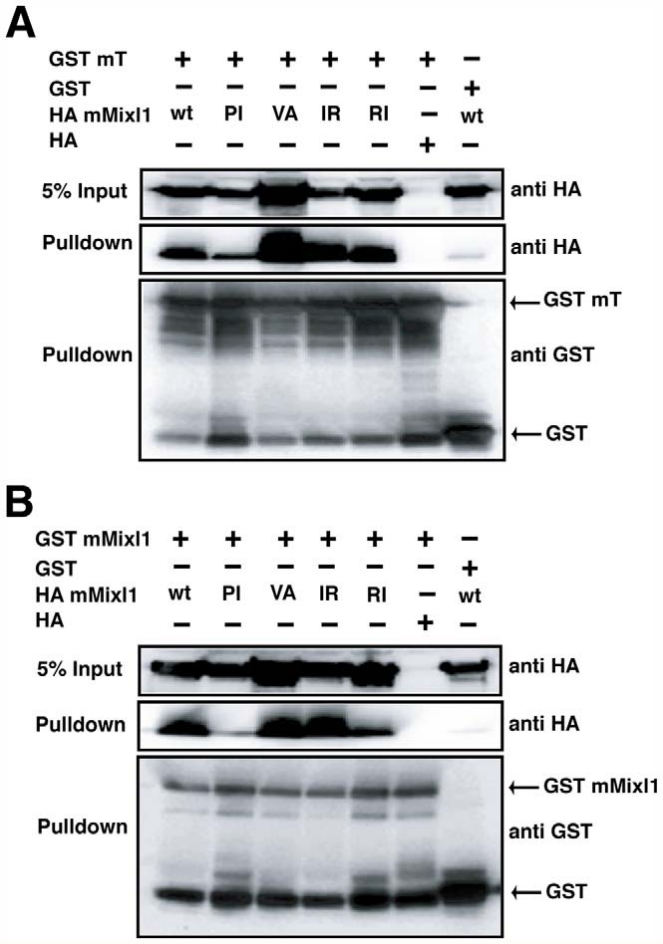
Mixl1 DNA binding activity is not required for its association with Brachyury. (A) Mixl1 DNA binding mutants and their interaction with T. 293T cells were transfected with GST mT and HA mMixl1 wt or Mixl1 HD DNA binding mutant expression plasmids as indicated in the text. GST-fusion proteins were isolated from whole cell extracts using glutathione resin and the bound fraction was analysed by Western blot with an anti-HA antibody. Expression of each protein was confirmed with anti-GST and anti-HA antibodies. (B) The Mixl1 P126I (PI) mutant shows reduced dimerisation. 293T cells were transfected with GST mMixl1 and HA mMixl1 wt and DNA binding mutant expression plasmids as indicated. Whole cell extracts were prepared and reactions performed as in (A).

Whilst all four Mixl1 HD mutants associated with GST-Brachyury, the P126I HD mutation showed reduced interaction with Brachyury (Figure 3A). When we examined this HD mutant for its ability to dimerise in solution with wt Mixl1, we observed that the P126I mutant displayed a markedly reduced dimerisation capacity (Figure 3B). These results suggest that Brachyury interacts with same domain within Mixl1 that is required for homodimerisation.

### Mixl1 interacts with additional members of the T-box family

**Figure 4:**
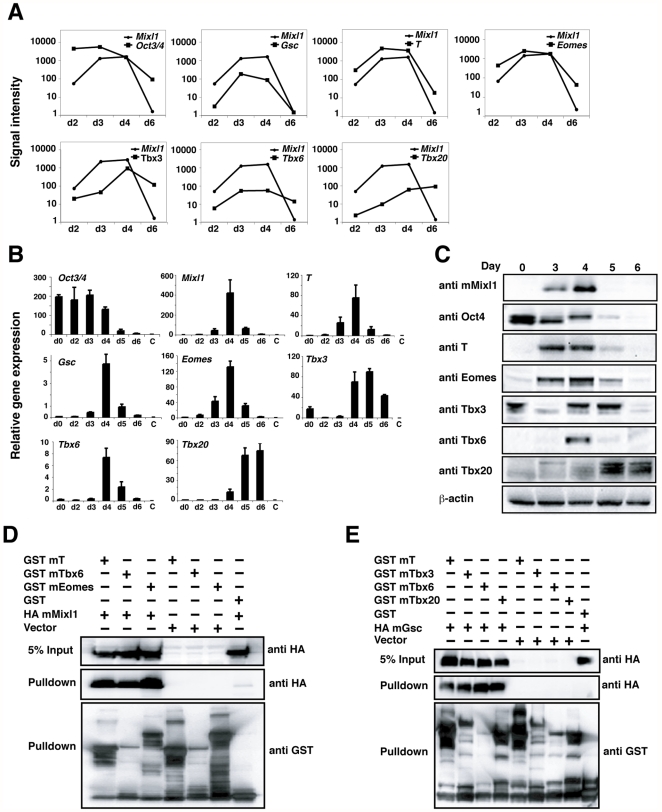
Tbx factors are co-expressed with Mixl1 during ESC differentiation and interact with Mixl1. (A) Signal intensity of *Mixl1*, *Oct3/4, Gsc* and *T* and the Tbx genes *Eomes, Tbx3, Tbx6* and *Tbx20* during ESC differentiation, as measured by microarray analysis. Expression profiles of the genes in (A) were validated by real time-PCR (B) results from an independent experiment are shown. Error bars show the S.E.M., n = 3 and Western blot analysis (C). (D) Mixl1 interacts with members of the Tbx family. 293T cells were transfected with HA mMixl1 and GST mT, mTbx6 or mEomes expression plasmids as indicated. GST-fusion proteins were isolated from whole cell extracts using glutathione resin and bound fractions were analysed by Western blot with anti-HA antibody. Expression of each protein was confirmed with anti-GST and anti-HA antibodies. (E) Gsc interacts with members of the Tbx family. 293T cells were transfected with HA mGsc and GST mT, mTbx3, mTbx6 or mTbx20 expression plasmids. Reactions were performed as in (D).

The T-box family contains several members that play important roles during embryonic development [19], [38]. We used Affymetrix GeneChip array analysis of differentiating *Mixl1* ^GFP/w^ mouse embryonic stem cells [37] to identify T-box factors whose expression overlapped that of *Mixl1* during ESC differentiation. Consistent with previous findings, the expression of *Mixl1* overlapped with the primitive streak gene *Gsc* and the stem cell marker *Oct4* [35], [39] (Figure 4A). We also observed that the expression of several members of the T-box family of genes including *Brachyury*, *Eomes*, *Tbx2*, *Tbx3*, *Tbx4*, and *Tbx6* largely overlapped with *Mixl1* at days 3 and 4 of ESC differentiation (Figure 4A and Figure S3). In the case of *Tbx20*, the overlap was partial (Figure 4A), whilst the expression profiles of *Tbx1*, *Tbx5*, *Tbx14* and *Tbx21* did not show substantial overlap with *Mixl1* expression (Figure S3). Real time PCR (Q-PCR) analysis provided independent confirmation that the T-box factors *Brachyury*, *Eomes*, *Tbx3*, and *Tbx6* were expressed during the transient wave of *Mixl1* transcription (Figure 4B). Western blot analysis of nuclear extracts collected from differentiating ESCs showed that protein expression for Mixl1, Brachyury, Eomes, Tbx3, Tbx6 and Tbx20 also correlated with their mRNA expression profiles (Figure 4C). Additionally, immunofluorescence showed co-expression of Eomes with Mixl1 in the nuclei of cells from d4 EBs (Figure S1B, C).

Therefore, we examined whether Mixl1 could also interact with other contemporaneously expressed T-box genes. We found that GST tagged versions of Eomes, Tbx3, Tbx6 and Tbx20 expressed in 293T cells could isolate Mixl1-T-box complexes (Figure 4D and Figure S4A). Consistent with our earlier observation that the Brachyury T-box domain could associate with Mixl1 (Figure 2B), we observed that the T-box domains of Eomes, Tbx3, Tbx6 and Tbx20 could also interact with Mixl1 (data not shown).

Given that expression of the paired-like homeodomain protein Gsc also overlapped with that of Mixl1 and T-box factors during ESC differentiation, we examined the potential for Gsc to complex with T-box factors (Figure 4A, B). These studies demonstrated that Gsc could indeed interact with T-box factors including Brachyury, Tbx3, Tbx6 and Tbx20 (Figure 4E). Conversely, we were unable to observe a stable interaction between Brachyury and POU-Homeodomain factors, including Oct4, whose expression also coincided with that of Mixl1 and Brachyury during ESC differentiation (Figure S4B). These results suggested that Mixl1 and the related paired-like homeodomain protein Gsc could physically interact with members of the T-box family of transcription factors.

### Mixl1 and Brachyury form a ternary complex on the Gsc MBS

**Figure 5:**
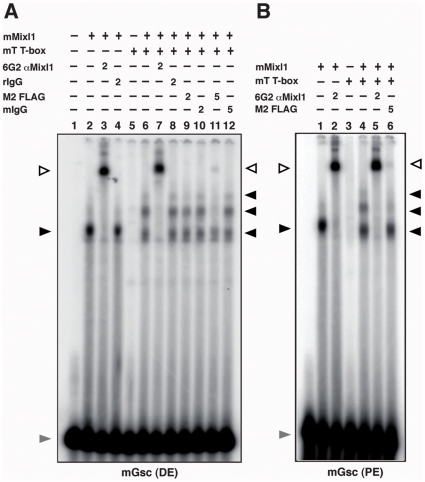
Interaction between T and DNA bound Mixl1. (A) Mixl1 and T form a complex on the *Gsc* promoter distal element (DE). EMSA was performed with *Gsc* promoter DE probe and 50 ng His-tagged Mixl1 protein (lanes 2 to 4 and 6 to 12). Samples in lanes 5 to 12 included 200 ng FLAG-His-tagged T T-box domain protein. The presence of Mixl1 and T in the DNA complexes was confirmed by super shift reactions using antibodies against Mixl1 (lanes 3 and 7) or the FLAG epitope (lanes 9 and 11). Lanes 3 and 7 received 2 µg 6G2 anti-Mixl1 antibody and lanes 4 and 8 received 2 µg rat isotype IgG. Lanes 9 and 11 received 2 µg and 5 µg of M2 FLAG antibody, showing that 5 µg of M2 FLAG antibody was required to demonstrate supershift activity. Lanes 10 and 12 received 2 µg and 5 µg of mouse IgG antibody, respectively. (B) Mixl1 and T form a complex on the *Gsc* promoter proximal element (PE). EMSA was performed as above except the *Gsc* PE MBS probe was used. Samples represented by lanes 1, 2 and 4 to 6 included 50 ng His-tagged Mixl1 protein whilst those present in lanes 3 to 6 contained 200 ng FLAG-His-tagged T T-box domain protein. Super shift reactions were performed by adding 6G2 anti-Mixl1 antibody (2 µg) to samples in lanes 2 and 5 or M2 FLAG antibody (5 µg) to the sample in lane 6. The black arrowheads indicate the position of the Mixl1 and Mixl1-T-DNA complexes; the white arrowhead shows the complexes super-shifted in the presence of the anti-6G2 or M2 FLAG antibodies; the grey arrowhead indicates the free probe.

We used electrophoretic mobility shift assays (EMSA) to determine the effect of Brachyury on the interaction between Mixl1 and promoter DNA. Mixl1 bound to *Gsc* promoter distal and proximal element Mixl1 binding site (MBS) probes as previously described (Figure 5) [6], [7] [37]. Addition of recombinant FLAG-tagged Brachyury T-box domain resulted in the formation of two slower-migrating complexes and a reduction in the intensity of the Mixl1-DNA complex. The presence of Mixl1 and Brachyury in the resulting complexes was confirmed by supershift experiments in which addition of anti-Mixl1 antibody [35] or FLAG M2 antibody, in a dose dependent manner, resulted in a supershift (Figure 5).

### T-box factors repress Mixl1 transactivation of the Gsc promoter

**Figure 6:**
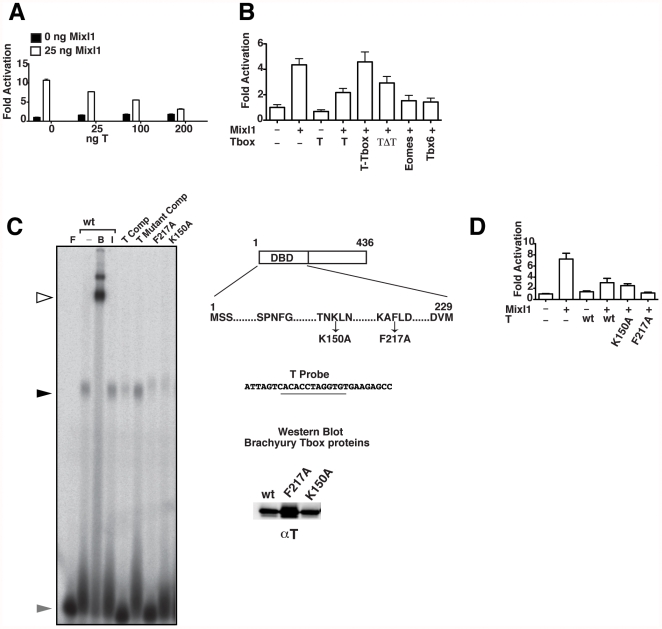
T and related Tbx factors reduce Mixl1 induction of the *Gsc* promoter. (A) Luciferase assays showing the effect of T on the transactivation activity of Mixl1. C2C12 cells were co-transfected with 100 ng of a *pGL3* Gsc promoter-luciferase reporter construct, 25 ng pMT2-HA-Mixl1 and increasing amounts of pMT2-HA-T as indicated. Regression analysis indicates that increasing amounts of T correlates with a significant decrease in Mixl1 transactivation, *p*<0.001. (B) The effect of T domains and related Tbx members on the transactivation activity of Mixl1. Luciferase Reporter analysis was performed as in (A) with 25 ng pMT2-HA-Mixl1 and 200 ng of pMT2-HA-T, T-Tbox, T ΔTbox (TΔT), Eomes or Tbx6. For A and B, results from an independent experiment are shown. Error bars show the S.E.M., n = 3, *p*<0.05 (Mixl1 vs Mixl1 co-transfected with T), *p*>0.05 (Mixl1 vs Mixl1 co-transfected with T-Tbox or TΔT), *p*<0.01 (Mixl1 vs Mixl1 co-transfected with Eomes or Tbx6). (C) The effect of T-box mutations on the DNA binding activity of T. EMSA was performed with a T DNA binding sequence probe and 50 ng His-tagged wt or mutant (K150A and F217A) T-box domain as indicated. A schematic of T showing the nature and position of mutations within the T-box domain is shown. The presence of T-box protein in the DNA complex was confirmed by super shift reactions using N19 anti-T antibody (lane B). Goat IgG was used as a control (lane I). Specificity of the T-box-DNA complex was confirmed by inclusion of a 100-fold molar excess of unlabeled T probe or mutant T probe competitor. The black arrowhead indicates the position of T-DNA complexes; the white arrowhead shows the complex super-shifted in the presence of anti-T antibody; the grey arrowhead indicates the free probe. The inset panel shows Western blot analysis using N19 anti-T antibody confirming the expression of wt and mutant T-box proteins. (D) Luciferase assay showing the effect of T mutants on the transactivation activity of Mixl1. Reporter analyses were performed on the *Gsc* promoter as in (A) with 50 ng pMT2-HA-Mixl1 and 200 ng of pMT2-HA-T or T mutants (K150A and F217A) as indicated. Results from an independent experiment are shown. Error bars show the S.E.M., n = 3 *p*<0.05 (Mixl1 vs Mixl1 co-transfected with wt T, or T mutants K150A and F217A).

To investigate the functional consequences of the Mixl1-Brachyury interaction, we used activation of transcription of the *Gsc* promoter as an assay for Mixl1 transcriptional activation [6], [7]. These experiments demonstrated that co-expression of Brachyury repressed the ability of Mixl1 to induce expression from the *Gsc* promoter in a dose dependent manner (Figure 6A). We examined the ability of Brachyury deletion mutants to modulate the action of Mixl1 on the Gsc promoter, to determine which domains within Brachyury were required for repression of Mixl1 transactivation activity. Unlike the full-length Brachyury protein, the Brachyury T-box domain alone (aa 1–229) did not repress Mixl1 induction of the *Gsc* promoter. Similarly a truncated Brachyury protein lacking the T-box domain (aa 230–436) did not repress Mixl1 transactivation activity (Figure 6B and Figure S5A). Analyses of additional T-box family members revealed that full length Eomes and Tbx6 also repressed the transactivation ability of Mixl1 on the *Gsc* promoter (Figure 6B and Figure S5A). Similar effects of Brachyury, Eomes and Tbx6 were also observed on the promoter of a second Mixl1 target gene, *Pdgfrα* (Figure S5B, C) [37].

Inspection of the *Gsc* promoter did not reveal consensus DNA binding sites for Brachyury [22], [23], suggesting that Brachyury was not directly binding to the *Gsc* promoter to regulate Mixl1 activity. Previous analysis of the structure of the *Xenopus* Brachyury T-box domain [40] revealed a conserved lysine residue within beta strand-*e* and a conserved phenylalanine residue located within helix H4 that were involved in DNA binding (Figure 6C). Therefore, in order to test if DNA binding was necessary for Brachyury to repress Mixl1 dependent transactivation, we constructed Brachyury mutants K150A and F217A and examined their ability to repress Mixl1 induction of the *Gsc* promoter (Figure 6C). Gel shift analysis showed that His-tagged recombinant wt Brachyury T-box domain bound to an oligonucleotide containing a previously determined consensus sequence for T-box factors (T probe) (Figure 6C) [22], [23]. Binding of Brachyury was confirmed by supershift analysis with an anti-Brachyury antibody and binding of Brachyury was specifically competed by unlabeled T probe (Figure 6C). In contrast, the K150A and F217A mutants displayed substantially reduced DNA binding activity (Figure 6C). Brachyury proteins possessing the K150A mutation repressed Mixl1 induction of the *Gsc* promoter at a level comparable to wt Brachyury, while the F217A mutant consistently displayed a stronger repressive effect when compared to wt Brachyury (Figure 6D and Figure S5D). This trend was also observed on the promoter of the *Pdgfrα* gene (Figure S5E). These results demonstrated that the DNA binding activity of Brachyury is not essential for the repression of Mixl1-mediated target gene transactivation.

## Discussion

The Mixl1 homeodomain transcription factor plays a key role in normal mesoderm and endoderm patterning during mammalian embryogenesis [12], [16]. Despite the wealth of information regarding the importance of Mixl1 during gastrulation, little is known about the molecular determinants underlying Mixl1 function. In particular the identity of protein cofactors and their effects on Mixl1 activity remains largely unknown. In this study, we addressed this issue and showed that members of the T-box family of transcription factors physically associated with Mixl1 and repressed its transactivation function on target gene promoters. For pragmatic reasons, we employed differentiating mouse ES cells as a model system to study early post-implantation embryonic development. Whilst there is sufficient evidence that findings in ES cells reflect events occurring in the embryo, we acknowledge that aspects of our in vitro model, especially the kinetics and the lack of complex structural organization within EBs might not accurately indicate events occurring during *in vivo* mammalian development.

Several sets of observations suggested that Brachyury and Mixl1 might regulate each other during mesoderm and endoderm formation. Firstly, in the early mouse embryo, Mixl1 and Brachyury are both expressed in the primitive streak [4], [41] and studies in *Xenopus* demonstrated cross repression by Mixl1 and Brachyury [3], [29], [42]. Similarly, constitutive expression of *Mixl1* during ESC differentiation repressed activin induced *Brachyury* expression, whilst analysis of *Mixl1*-null ESC lines and mice revealed that loss of *Mixl1* resulted in an up-regulation of *Brachyury* expression in vitro [37] and in vivo [16]. These results were also consistent with reports in *Xenopus* where *Mix.1* down regulated *Xbra* expression, in part through the activation of *Goosecoid* [3], [29], [42]. In RNAi-mediated knockdown (KD) experiments in ESCs, *Mixl1* KDs resulted in enhanced Brachyury expression whilst *Mixl1* overexpression suppressed Brachyury expression [30]. The results presented in our study extend the scope of this previous work, suggesting that a functional relationship in which Brachyury represses Mixl1 might be based on their physical interaction.

We provide several lines of evidence to support this notion. Firstly, by immunofluorescence analysis, we observed the presence of Mixl1^+^Brachyury^+^ cells at d4 of differentiation. Second, through co-immunoprecipitation and GST-pulldown experiments, we showed that Mixl1 physically associated with Brachyury and that this interaction was conserved in several vertebrate species. Others have demonstrated physical associations between homeobox proteins and T-box family members [43]–[47]. Our mapping studies demonstrated that the T-box domain of Brachyury and the Mixl1 homeodomain were important domains for interaction, consistent with previous findings documenting interactions between the T-box and homeodomains [43], [47].

While our data suggested important roles for the Mixl1 and Brachyury DNA binding domains, we also provide evidence that other regions may contribute to the interaction, supporting a model where multiple domains within Mixl1 and Brachyury underpin their association. This conclusion mirrors previous studies showing that sequences outside the T-box domain of Tbx5 contributed to its interaction with the homeodomain protein, Nkx2-5 [43]. An important finding was that Mixl1 mutants defective for DNA binding [2], [5], [11] were still able to interact with Brachyury. An exception was the Mixl1 mutant (P126I) that displayed substantially reduced binding to Brachyury. Strikingly we observed that this mutation also impaired the ability of Mixl1 to associate with itself, presumably as a homodimer. These results suggest a number of interesting possibilities. Firstly, since the capacity of Mixl1 to bind DNA is not a pre-requisite for it to interact with Brachyury, part of the pool of Mixl1 and Brachyury within the cell may exist as a pre-formed complex sequestered from DNA. These factors might then be recruited alone or as a complex to promoter DNA in order to regulate gene transcription. Second, our data suggests that helix 3 of the Mixl1 HD is a direct interaction surface for Brachyury or that Brachyury has a preference to associate with Mixl1 homodimers. Further structural analysis of the Mixl1-Brachyury complex may shed further light on the function and dynamics of the Mixl1-Brachyury interaction.

*Brachyury* is the founding member of a family comprising at least 18 mammalian T-box genes that are involved in the induction and regional specification of mesoderm [19], [24], [48]. We provide evidence to support the notion that in addition to Brachyury, other members of the T-box family might interact with Mixl1. Firstly, we confirmed that the expression of the T-box genes *Eomes*, *Tbx2*, *3*, *6* and *20* significantly overlapped with the transient expression of *Mixl1* at days 3 and 4 of ESC differentiation. Furthermore, immunofluorescence analysis of differentiating EBs revealed the presence of a Mixl1^+^Eomes^+^ population of cells during ESC differentiation. The expression pattern of these genes is consistent with their previously reported expression in the primitive streak and emerging mesoderm of gastrulation stage embryos [48]–[50]. Second, our biochemical analyses demonstrated that Eomes, Tbx2, 3, 6 and 20 were all capable of interacting with Mixl1. Our interaction studies suggested that these same four T-box factors could also interact with the related homeodomain protein, Gsc [51]–[53]. The finding that multiple members of the T-box family bind Mixl1 or Gsc suggests that the Mixl1-Brachyury interaction reflects a generic propensity for association between paired homeobox proteins and T-box factors. Notably, we were unable to detect an interaction between Brachyury and the POU-homeodomain factors Oct4 or Oct6, suggesting that additional sequence requirements govern Mixl1-T-box interactions and that not all classes of homeodomain proteins associate with T-box factors.

The functional significance of the Mixl1-Brachyury association was demonstrated in luciferase reporter experiments in which Brachyury repressed Mixl1 activation of the *Gsc* promoter. A similar effect was observed with Eomes and Tbx6. Whilst it was possible that Brachyury would block Mixl1 binding to DNA, we observed that the T-box domain of Brachyury actually formed a ternary complex with DNA-bound Mixl1. These observations suggested that Brachyury and related T-box factors might be recruited to target genes via the association of their T-box domain with promoter bound Mixl1. Such an arrangement might allow T-box factors to regulate genes to whose promoters they do not directly bind. Our observation that non-DNA binding mutants of Brachyury repressed Mixl1 activity and that Brachyury alone did not transactivate the *Gsc* promoter are consistent with this idea. This indirect mode of promoter repression through complex formation with an unrelated transactivating factor has previously been described for basic helix-loop-helix factors such as Hey1 protein and its association with the GATA family of transcriptional activators [54].

**Figure 7:**
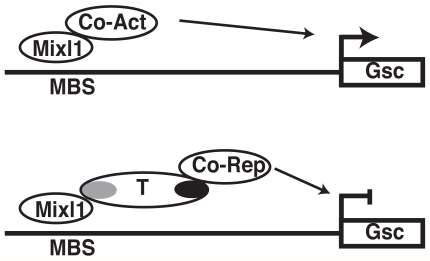
Model for repression of Mixl1 transactivating ability by T. Top panel: Mixl1 binds and activates expression of the *Gsc* gene promoter through the recruitment of co-activators. Bottom panel: Mixl1 recruits T through the association of the HD with the T T-box domain (grey). The carboxy terminal portion of T (black) mediates recruitment of co-repressors at the *Gsc* gene, resulting in gene repression.

The repressive effect of Brachyury might be mediated through the recruitment of co-repressor complexes to the *Gsc* promoter (Figure 7). While Brachyury has largely been described to function as a transcriptional activator [22], [23], [55], mapping of regulatory domains in the carboxy terminal half of Brachyury has identified repression domains [23]. In addition, Tbx6 has been shown to repress gene expression [56]. More recently, the T-box proteins Tbx15 and Tbx18 have been suggested to repress promoter activity through the recruitment of Groucho/HDAC or CtBP/HDAC repressor complexes [45] while a C terminal motif in Tbx2 and Tbx3 mediates repression by direct association with HDAC1 [57]. It is noteworthy that in our reporter assays, neither the Brachyury T-box domain nor the carboxy-terminal portion alone repressed Mixl1 transactivation activity. These observations lead us to speculate that the T-box, in this context, might target Tbx proteins to promoter bound homeodomain factors while the carboxy-terminal region participates in the recruitment of co-repressor factors (Figure 7).

In summary, these studies represent the first demonstration that the paired-like homeodomain protein Mixl1 can interact with several members of the Tbx protein family. These data raise the interesting possibility that the temporal and/or spatial expression of Tbx factors during development may influence the Tbx partner choice and transcriptional activity of Mixl1. Future experiments aimed at establishing which Mixl1-Tbx interactions take place during the establishment and patterning of the primary germ layers will help shed light on the function of Mixl1-Tbx complexes during early development.

## Materials and Methods

### Plasmids

p*EF-BOS* FLAG *Mixl1* was created by cloning a PCR fragment generated from *Mixl1* cDNA into the *Asc*I site of p*EF-BOS* [58]. pMT2-HA Mixl1 and pMT2-HA Goosecoid (*Gsc*) were created by cloning PCR fragments generated from *Mixl1* and *Gsc* cDNAs into the *Sal*I and *EcoR*I sites of pMT2-SM HA. pMT2-HA Brachyury (*T*) was created by cloning a Brachyury cDNA into the *Xho*I and *EcoR*I sites of pMT2-SM HA. pMT2-HA Tbx6 was created by cloning a Tbx6 cDNA into the *Not*I and *SalI* sites of pMT2-SM HA. pMT2-HA Eomoseodermin (*Eomes*) was created by cloning a *Eomes* cDNA into the *Xho*I and *EcoR*I sites of pMT2-SM HA. pMT2-SM HA containing the T-box domain of Brachyury was created by cloning a cDNA encompassing nt 1–687 of Brachyury into the *XhoI* site of pMT2-SM HA. pMT2-SM HA ΔT-box-Brachyury was created by cloning a cDNA excluding nt 1–687 of mBrachyury into the *Xho*I and *EcoR*I sites of pMT2-SM HA. To ensure nuclear localization of the Brachyury T-box and ΔT-box-Brachyury proteins an SV40 nuclear localization sequence (PKKKRKV) [59] was cloned between the *Not*I/*SalI* sites and *Not*I/*XhoI* sites upstream of the cDNAs for pMT2-SM HA Brachyury T-box and pMT2-SM HA ΔT-box-Brachyury, respectively. pMT2-HA Oct4 and Oct6 were created by cloning cDNAs spanning the coding regions of each gene into the *Xho*I and *EcoR*I sites (for Oct4) and the *Sal*I and *EcoR*I sites (for Oct6) of pMT2-SM HA. To obtain N-terminally fused GST-tagged proteins, *Mixl1*, *Brachyury*, *Tbx3*, *Tbx6*, *Tbx20* and *Eomes* cDNA sequences were generated by PCR from a day 4 differentiated *Mixl1^GFP^* ^/*w*^ ESC [13] cDNA library, and cloned into the pDONR221 entry vector via the BP reaction (Gateway Technology, Invitrogen). pENTRY clones were subsequently used in LR reactions (Invitrogen) with the expression vector pDEST27 (GST). Sequences encoding truncated Mixl1 or Brachyury were made from cDNA using a PCR based approach and cloned into pDONR221 via the BP reaction (Invitrogen). pENTRY clones were subsequently used in LR reactions (Invitrogen) with the mammalian expression vector pDEST27 (GST). Similarly for bacterial protein expression, GST-mMixl1, GST-hMixl1, GST-xMixl1, His-Brachyury, His-Brachyury T-box domain, His-Brachyury and His-Xbra derivatives were constructed by Gateway cloning using Gateway converted bacterial expression vectors pGEX2T (GST) or pET15b (His). Site directed mutagenesis of the Brachyury T-box domain (K150A and F217A) was performed with the pET15b Brachyury T-box construct using the Gene Tailor site directed mutagenesis system according to the manufacturer's instructions (Invitrogen). *pGL3* reporter constructs contained the luciferase gene linked to genomic fragments from *Gsc* (111 bp) or *Pdgfrα* (1 kbp) promoters. These genomic fragments represented sequences immediately upstream from the transcription start site in the case of *Pdgfrα* or nts-574 to -463 relative to the transcription start site in the case of the *Gsc* promoter.

### Quantitative Real-Time Polymerase Chain Reaction

RNA from undifferentiated and differentiated ESCs was prepared using the RNAeasy kit according to the manufacturer's instructions (Qiagen). First strand cDNA was reverse transcribed with random hexamer priming using Superscript III reagents (Invitrogen). Quantitative real-time polymerase chain reaction was performed using TaqMan® gene expression probes and Taqman® reagents (Applied Biosystems) and the 7500 Fast Real-Time PCR System absolute thermal cycler and software (Applied Biosystems). Quantification studies using TaqMan® Gene Expression Assays were carried out according to the manufacturer's instructions with the following TaqMan® Gene Expression Assay probe sets: *Tbx3* (Mm01195726_m1); *Tbx6* (Mm00441681_m1); *Tbx20* (Mm00451515_m1); *Eomes* (*Tbr2*) (Mm01351985_m1); *Brachyury* (Mm00436877_m1); *Mixl1* (Mm00489085_m1); *Goosecoid* (Mm00650681_g1); *Oct4* (Mm00658129_gH) and *Hprt* (Mm00446968_m1). For each of the gene specific primers sets used, the signal was compared to that obtained with *Hprt* and the results expressed as a relative gene expression as described previously [60].

### ESC growth and differentiation

The Mixl1*^w^* ^/*w*^ (W9.5) [61], heterozygous *Mixl1^GFP/w^* [13] and homozygous null *Mixl ^GFP/Hygro^* ESCs [37] were cultured on primary mouse embryonic fibroblast (PMEF) feeder layers as described [62] in ESC media (high glucose DMEM supplemented with 15% FCS (v∶v) and 1000 U/ml Leukaemia Inhibitory Factor (LIF). ESCs were differentiated as embryoid bodies as described [13]. On the day prior to differentiation, ESCs were passaged onto fresh PMEFs. ESCs and PMEFs were harvested and re-suspended in Iscove's Modified Dulbecco's Medium (IMDM) (Invitrogen) supplemented with 10% FCS. Cells were then transferred to a non-gelatinised tissue culture dish and placed at 37°C in an incubator for 40 minutes to separate PMEFs from ES cells by virtue of their more rapid adherence. The non adherent ES cell fraction was harvested and disaggregated ES cells seeded at 5000 cells/ml in 6 cm non-adherent dishes in differentiation medium, comprising IMDM supplemented with 15% FCS (v∶v), 5% protein free hybridoma medium II (v∶v) (Invitrogen), 2 mM glutamine (v∶v), 50 µg/ml ascorbic acid (w∶v) (Sigma) and 4.5×10^−4^ M α-MTG (Sigma). Embryoid bodies formed and grew for the indicated number of days. Cultures were maintained at 37°C in a humidified environment of 8% CO_2_ in air. The initiation of differentiation was denoted as day 0.

### Cell culture and luciferase assays

293T (ATCC CRL-11268) and C2C12 (ATCC CRL-1772) cells were maintained in Dulbecco's modified Eagle's medium supplemented with 10% fetal bovine serum. C2C12 cells were transfected using FuGENE 6 reagent (Roche) and assayed for luciferase activity as previously described [6]. Reporter and expression plasmids were added in the amounts indicated in the figure legends. Transfection of 50 ng pRTKluc (Renilla) served as a transfection control and was used to normalize luciferase activity. All data shown represents an average of at least three experiments performed in triplicate. Error bars represent standard error of the mean where n = 3.

### Double indirect immunofluorescence cell staining

Day 4 and day 9 W9.5 EBs were fixed in 4% (w/v) paraformaldehyde (PFA) in 10 mM PBS (pH 7.2) for 20 minutes and 40 minutes at room temperature, respectively. Following fixation, samples were rinsed twice in PBS. Aggregates of day 4 and day 9 mouse EBs were then pelleted, covered in 0.7% (w/v) low melt agarose, paraffin embedded and subsequently sectioned at 5 µm. Following de-waxing, histological sections underwent an antigen retrieval step followed by brief washes in deionised water and PBS. Sections were then washed in PBS containing 0.5% bovine serum albumin, 0.1% Tween 20 and 5% donkey serum at room temperature for 30 min and then labelled overnight at 4°C with Brachyury N-19 (diluted 1∶50, Santa Cruz Biotechnology). A negative control consisted of the parallel staining step on day 9 mouse EB sections. Anti-Brachyury was detected by incubation with biotinylated donkey anti-goat IgG (diluted 1∶100, Vector Laboratories), for 30 minutes at room temperature followed by Streptavidin Alexa Fluor® 488 (diluted 1∶500, Molecular Probes). Sections were blocked with goat serum diluted 1∶20 in 0.5% BSA/PBS-T and incubated with anti-Mixl1 2D10 rat monoclonal antibody (diluted 1∶50) [35] for 1 hour at room temperature. Anti-Mixl1 was detected by incubation with Alexa Fluor® 568 goat anti-rat IgG (diluted 1∶100, Molecular Probes). All sequential antibody incubation steps included three washes in PBS containing 0.1% Tween 20. In case of Mixl1 and Eomes staining, sections were incubated for 30 minutes in normal goat serum diluted 1∶20 in PBS containing 0.1% Tween 20 and 0.5% BSA (0.5% BSA/PBS-T) and then labeled overnight at 4°C with anti-Mixl1 2D10 rat monoclonal antibody (diluted 1∶50) [35] and an anti-Eomes rabbit polyclonal antibody (diluted 1∶50, Chemicon International). Negative controls consisted day 9 mouse EBs processed in parallel. Sections were then incubated with biotinylated goat anti-rabbit IgG (diluted 1∶100, Vector Laboratories) for 30 minutes at room temperature. Anti-Mixl1 and anti-Eomes were detected by incubation with Alexa Fluor® 568 goat anti-rat IgG (diluted 1∶100, Molecular Probes) and Streptavidin Alexa Fluor® 488 (diluted 1∶500, Molecular Probes). All sequential antibody incubation steps included three washes in PBS containing 0.1% Tween 20. Sections were incubated in the nuclear stain (TOPRO-3 diluted 1∶500, Molecular Probes) for 5 minutes at room temperature and mounted with anti-fade mounting medium. Confocal images were captured using the Leica TCS SP-5 confocal microscope.

### Recombinant protein expression and purification

*E. coli* strain BL21 RIPL(DE3) (Stratagene) containing pGEX-mMixl1, pGEX-hMixl1, pGEX-xMixl1, pET15b-mBrachyury, pET15b-mBrachyury T-box domain, pET15b-hBrachyury or pET15b-Xbra derivatives was cultured at 37°C in 2.5 litre LB media containing ampicillin (100 µg/ml) and chloramphenicol (30 µg/ml). At an OD_600_ of 0.5–0.6 the cells were induced with IPTG (1.0 mM). After 5 h at 30°C cells were harvested and the cell pellet resuspended in 30 ml of lysis buffer (50 mM Tris-HCl pH 8.0, 20% sucrose, 1 mM EDTA, 0.3 M KCl, 0.5% Triton X-100, 1 mM DTT, 0.5 mM PMSF, 1 µg/ml Aprotinin and 1 µg/ml Leupeptin). The cells were incubated on ice with 200 µg/ml lysozyme, lysed by freeze-thawing and sonication and the bacterial lysate cleared by ultracentrifugation. His-Brachyury derivatives were batch purified by mixing clarified lysates containing 10 mM Imidazole with Talon resin (Clontech) for 4 h at 4°C with gentle rotation. The slurry was washed three times in wash buffer (50 mM Tris-HCl pH 8.0, 150 mM KCl, 0.5% Triton X-100, 10 mM Imidazole, 0.5 mM PMSF, 1 µg/ml Aprotinin and 1 µg/ml Leupeptin) and the His-Brachyury proteins were eluted in wash buffer containing 300 mM Imidazole for 30 min at 4°C with gentle rotation. The preparations were dialysed against ELB_150_ buffer (50 mM HEPES-KOH pH 7.9, 150 mM KCl, 5 mM MgCl_2_, 0.5 mM EDTA, 0.5% TX-100, 0.5 mM PMSF, 1 µg/ml Aprotinin and 1 µg/ml Leupeptin). GST-Mixl1 derivatives were batch purified by mixing clarified lysates with glutathione agarose (Sigma) for 4 h at 4°C with gentle rotation. The slurry was washed three times in wash buffer (50 mM Tris-HCl pH 8.0, 150 mM KCl, 1 mM EDTA, 0.5% Triton X-100, 0.5 mM PMSF, 1 µg/ml Aprotinin and 1 µg/ml Leupeptin) and the GST-Mixl1 proteins were eluted in wash buffer containing 15 mM Glutahione for 30 min at 4°C with gentle rotation. The preparations were dialysed against ELB_150_ buffer.

### Preparation of nuclear extracts

Differentiating W9.5 ES cell nuclear extracts were prepared as previously described [63] and dialysed against ELB_150_ buffer.

### Protein-Protein interaction analyses

To perform co-immunoprecipitation assays, 293T cells were transfected with pEF-BOS FLAG or pMT2-SM HA expression vectors encoding Mixl1, Goosecoid, Oct4 or Brachyury proteins in combinations indicated in the figure legends using FuGENE 6 reagent as described by the manufacturer (Roche). After transfection (72 hours) cells were lysed in ELB_150_ buffer. For co-immunoprecipitation, 293T cell lysates were incubated in ELB_150_ buffer with 5 µg anti-FLAG M2 antibody or 5 µg isotype control antibody with rotation at 4°C. After 4 h, pre-washed protein G-beads were added to recover immunoprecipitates, washed in ELB_150_ buffer and analysed by Western blot analysis. For GST pull-down experiments using mammalian cell extracts, 293T cells were transfected as above with pDEST27 and pMT2-SM HA expression vectors encoding Mixl1 or Brachyury proteins in combinations indicated in the figure legends. After transfection (72 h), cells were lysed in ELB_150_ buffer. 293T cell lysates were incubated in ELB_150_ buffer with pre-washed glutathione agarose (Sigma) with rotation at 4°C, washed in ELB_150_ buffer and subjected to Western blot analysis. To analyse interactions between bacterial produced Mixl1 and Brachyury, purified lysates containing His-Brachyury derivatives were incubated with purified lysates containing equivalent levels of GST-Mixl1 derivatives or GST alone and 100 µl of pre-washed glutathione beads in 500 µl of ELB_150_ buffer. Reactions were performed at 4°C for 3 h with rotation. Complexes were washed in ELB_300_ buffer containing 0.3 M KCl and subjected to Western blot analysis.

### Protein-DNA interaction assays

Electrophoretic mobility shift assays (EMSA) were performed with 5000 c.p.m. of ^32^P-labeled double-stranded P3, Gsc DE MBS, Gsc PE MBS or Brachyury T DNA probes as previously described [6].

### Antibodies and Western blotting

Protein extracts were resolved on 4–12% Bis-Tris gels and transferred to PVDF membrane. Membranes were blocked with Superblock Blocking Buffer (Pierce) and subsequently probed with the following antibodies: anti-6G2 [35], anti-Brachyury (Santa Cruz, N19), anti-GST (Santa Cruz, B14), anti-HA (Roche), anti-actin (Sigma), anti-Tbr2 (Eomes; Chemicon), anti-Tbx6 (abcam, 35733), anti-Tbx3 (Santa Cruz, S17), anti-Tbx20 (abcam, 42468) and anti-Oct4 (Santa Cruz, N19). Primary antibodies were detected using a Chemiluminescence Substrate Kit according to the manufacturer's instructions (GE).

### Microarray analysis

*Mixl1* and *Tbx* gene expression profiles were obtained from Affymetrix® array gene profiling experiments [37]. Primary data is available through (http://www.ebi.ac.uk/arrayexpress/) via Accession number E-MEXP-1976.

## Supporting Information

Figure S1 **Immunofluorescence analysis of differentiated W9.5 ESCs.** (A) Immunofluorescence analysis of Mixl1 and Brachyury expression in day 9 differentiated W9.5 ESCs. In contrast to d4 immunofluorescence images presented in Figure 1, staining with anti-T and anti-Mixl1 antibodies at day 9 did not reveal expression of either protein. Nuclei were visualized with TOPRO (Blue). Original magnification: ×50. (B) Mixl1 protein is co-expressed with Eomes. Immunofluorescence analysis of day 4 differentiated W9.5 ESCs showing co-expression of Eomes (green) and Mixl1 (red) proteins. Nuclei were visualized with TOPRO (Blue). Original magnification: ×50 upper row and ×100 lower row. (C) As a negative control, day 9 W9.5 ESCs were subjected to the same staining protocol as in (B). No specific antibody staining was observed at this time. Nuclei were visualized with TOPRO (Blue). Original magnification: ×50 upper row and ×100 lower row.
(TIF)

Figure S2 **Association of Mixl1 with homeodomain proteins.** 293T cells were transfected with FLAG mMixl1 together with HA mMixl1 (A), HA mGsc (B) or HA mOct4 (C) as indicated. Whole cell lysates were subjected to immunoprecipitation (IP) with anti-FLAG antibody or IgG control antibody followed by Western blot analysis with anti-HA antibodies.
(TIF)

Figure S3 **Expression of Tbx factors during ESC differentiation.** Graphs showing the signal intensity of *Mixl1*, and the Tbx genes *Tbx1, Tbx2, Tbx4, Tbx5, Tbx14* and *Tbx21* detected by microarray analysis of EBs from d2 to d6 of differentiation.
(TIF)

Figure S4 **Mixl1 associates with Tbx proteins.** (A) 293T cells were transfected with HA mMixl1 together with GST mT, mTbx3, mTbx6 or mTbx20 as indicated. Whole cell extracts were prepared and the GST-fusion proteins isolated using glutathione resin. Bound fractions were analysed by Western blot analysis with an anti-HA antibody. Expression of each protein was confirmed with anti-GST and anti-HA antibodies. (B) Analysis of the interaction between T and Oct4 and Oct6. 293T cells were transfected with GST-mT together with HA-mMixl1, HA-mOct4 or HA-mOct6. Whole cell extracts were prepared and the GST-fusion proteins were isolated using glutathione resin. Bound fractions were analysed by Western blot analysis with an anti-HA antibody. Expression of each protein was confirmed with anti-GST and anti-HA antibodies.
(TIF)

Figure S5 **T and related Tbx factors reduce Mixl1 induction of the** ***Gsc*** **and** ***Pdgfrα*** **promoters.** (A) An additional replicate of the luciferase reporter assays showing the effect of T domains and related Tbx members on the transactivation activity of Mixl1. Luciferase reporter analysis was performed on the *Gsc* promoter with 25 ng pMT2-HA-Mixl1 and 200 ng of pMT2-HA-T, T-Tbox, T ΔTbox (TΔT), Eomes or Tbx6. Results from an independent experiment are shown. Error bars show the S.E.M., n = 3. (B) Luciferase reporter analysis was performed on the *Pdgfrα* promoter with 25 ng pMT2-HA-Mixl1 and increasing amounts of pMT2-HA-T. Results from an independent experiment are shown. Error bars show the S.E.M., n = 3. (C) Luciferase reporter analyses were performed on the *Pdgfrα* promoter as outlined in (B) with 25 ng pMT2-HA-Mixl1 and 200 ng of pMT2-HA-T, T-Tbox, T ΔTbox (TΔT), Eomes or Tbx6. Results from two independent experiments are shown. Error bars show the S.E.M., n = 3. (D) Additional replicates of the luciferase assay showing the effect of T mutants on the transactivation activity of Mixl1. Reporter analyses were performed on the *Gsc* promoter with 50 ng pMT2-HA-Mixl1 and 200 ng of pMT2-HA-Brachyury or pMT2-HA-Brachyury T-box mutants K150A and F217A. Results from two independent experiments are shown. Error bars show the S.E.M., n = 3. (E) Luciferase reporter analyses were performed on the *Pdgfrα* promoter as outlined in (D). Results from three independent experiments are shown. Error bars show the S.E.M., n = 3.
(TIF)
