# Supplementary figures and images for: Brachyury and Related Tbx Proteins Interact with the Mixl1 Homeodomain Protein and Negatively Regulate Mixl1 Transcriptional Activity
Source: PLoS One. 2011 Dec 2;6(12):e28394. doi: 10.1371/journal.pone.0028394 (PMC3229578; doi:10.1371/journal.pone.0028394)

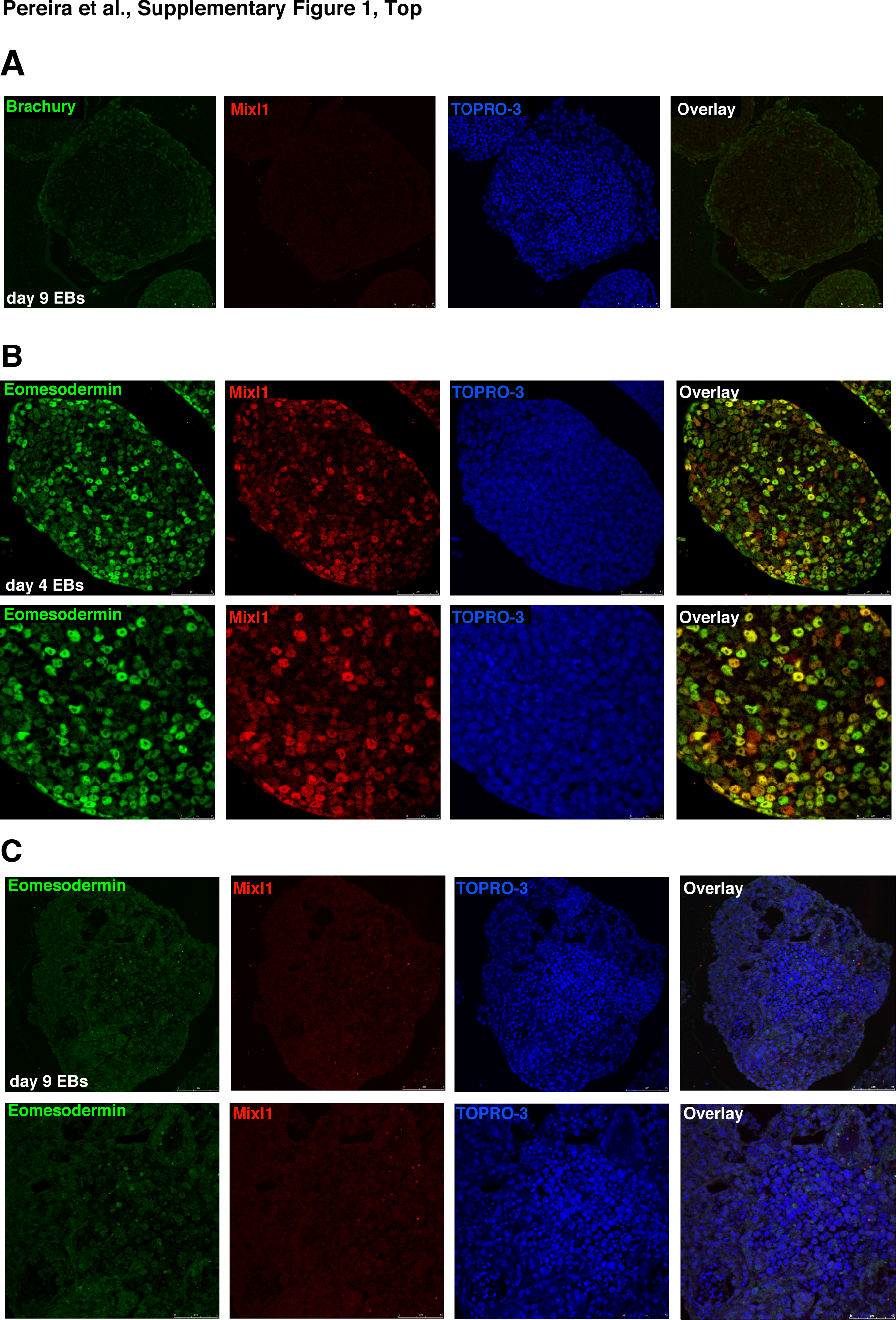

Supplement: Figure S1 — Immunofluorescence analysis of differentiated W9.5 ESCs. (A) Immunofluorescence analysis of Mixl1 and Brachyury expression in day 9 differentiated W9.5 ESCs. In contrast to d4 immunofluorescence images presented in Figure 1, staining with anti-T and anti-Mixl1 antibodies at day 9 did not reveal expression of either protein. Nuclei were visualized with TOPRO (Blue). Original magnification: ×50. (B) Mixl1 protein is co-expressed with Eomes. Immunofluorescence analysis of day 4 differentiated W9.5 ESCs showing co-expression of Eomes (green) and Mixl1 (red) proteins. Nuclei were visualized with TOPRO (Blue). Original magnification: ×50 upper row and ×100 lower row. (C) As a negative control, day 9 W9.5 ESCs were subjected to the same staining protocol as in (B). No specific antibody staining was observed at this time. Nuclei were visualized with TOPRO (Blue). Original magnification: ×50 upper row and ×100 lower row. (TIF) [file pone.0028394.s001.tif]

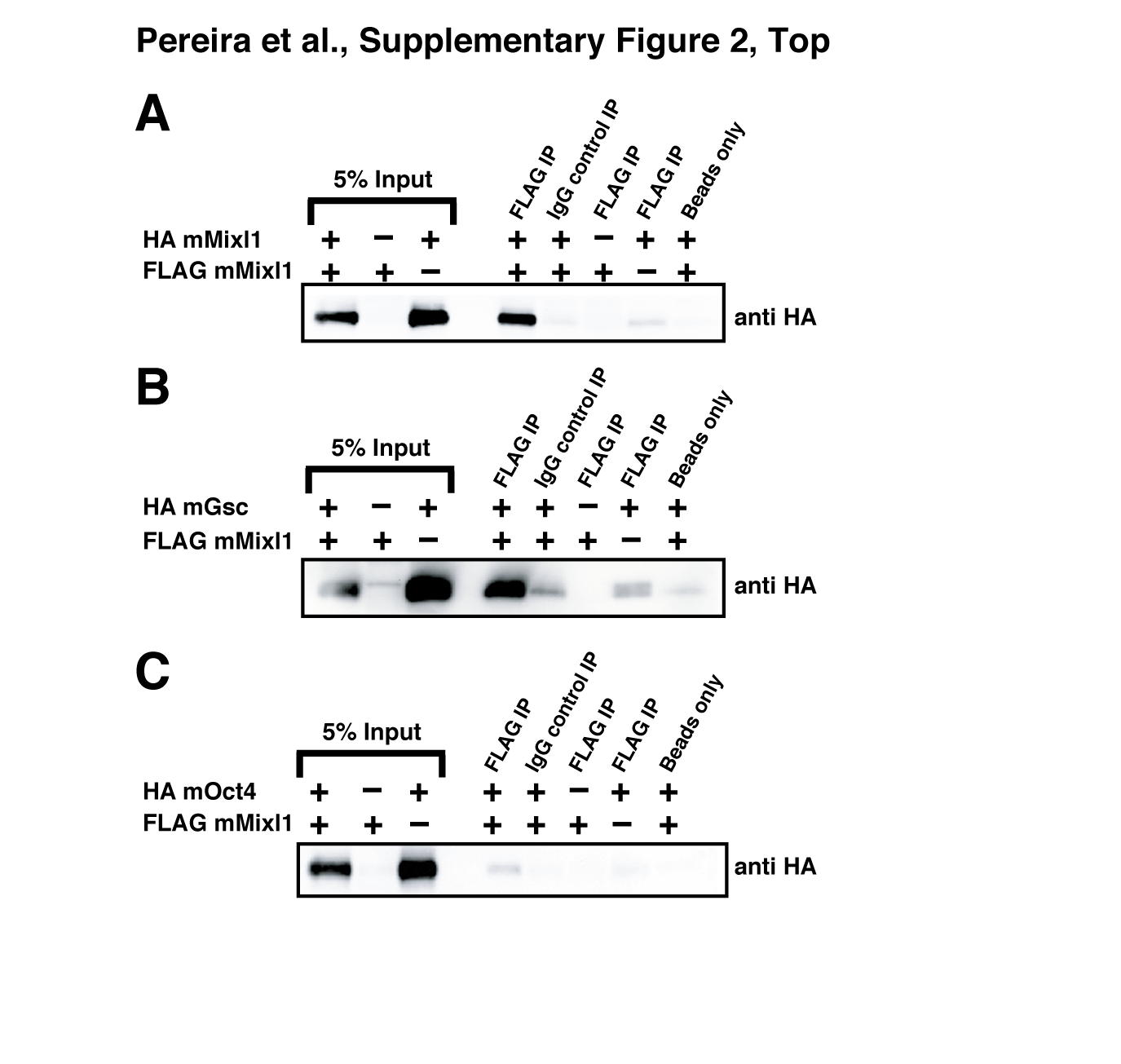

Supplement: Figure S2 — Association of Mixl1 with homeodomain proteins. 293T cells were transfected with FLAG mMixl1 together with HA mMixl1 (A), HA mGsc (B) or HA mOct4 (C) as indicated. Whole cell lysates were subjected to immunoprecipitation (IP) with anti-FLAG antibody or IgG control antibody followed by Western blot analysis with anti-HA antibodies. (TIF) [file pone.0028394.s002.tif]

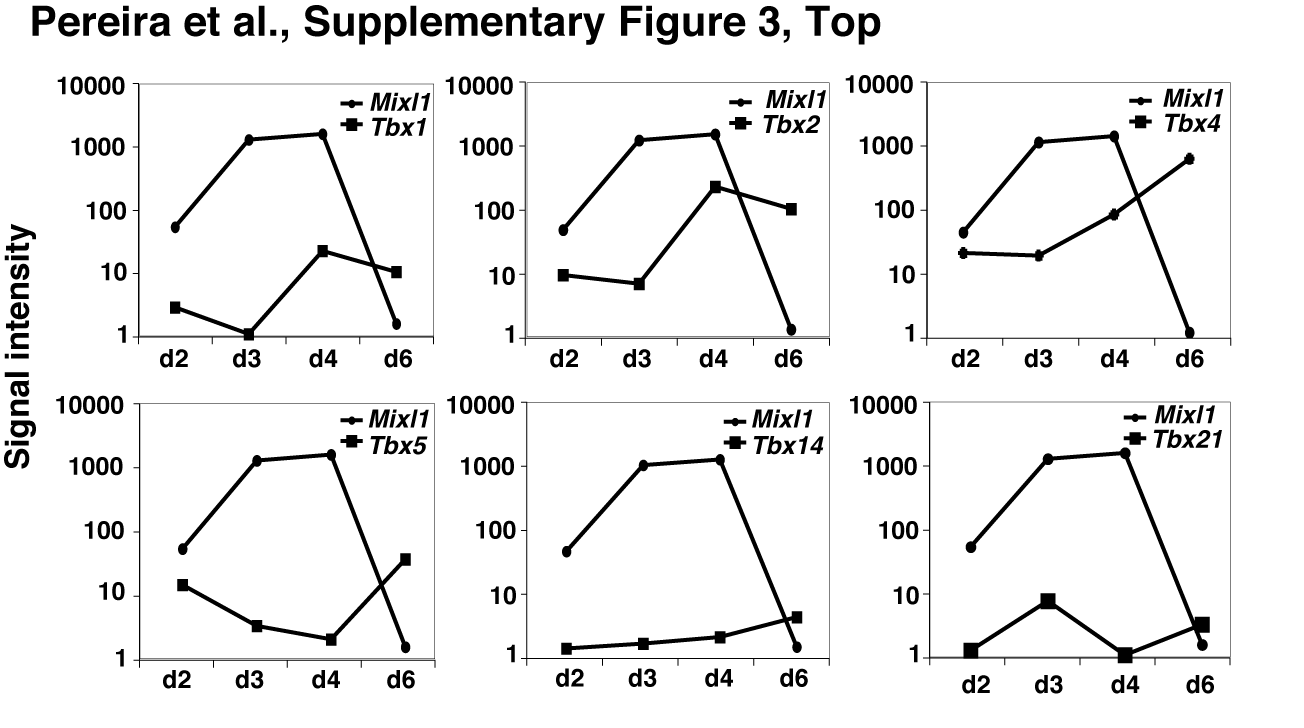

Supplement: Figure S3 — Expression of Tbx factors during ESC differentiation. Graphs showing the signal intensity of Mixl1, and the Tbx genes Tbx1, Tbx2, Tbx4, Tbx5, Tbx14 and Tbx21 detected by microarray analysis of EBs from d2 to d6 of differentiation. (TIF) [file pone.0028394.s003.tif]

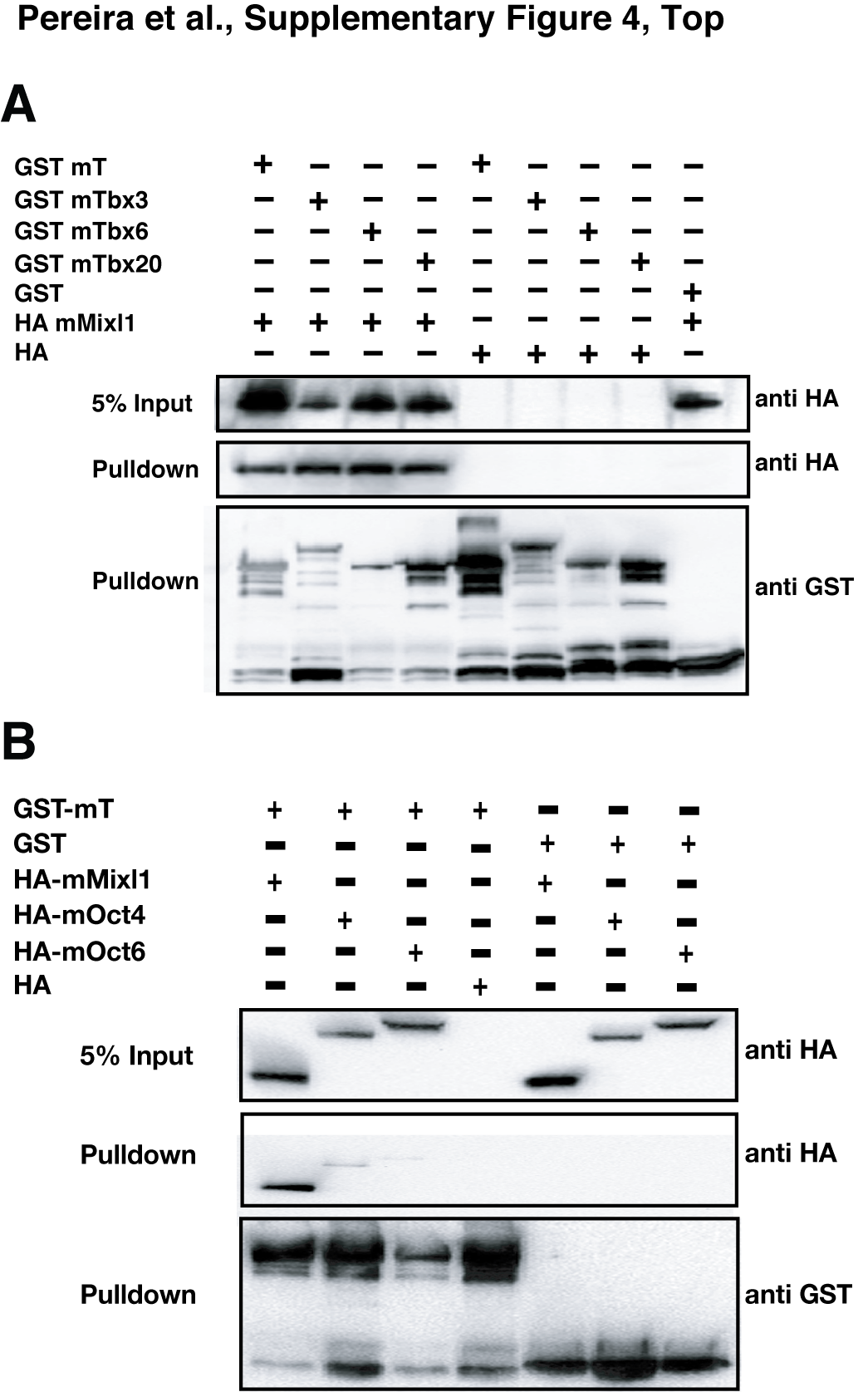

Supplement: Figure S4 — Mixl1 associates with Tbx proteins. (A) 293T cells were transfected with HA mMixl1 together with GST mT, mTbx3, mTbx6 or mTbx20 as indicated. Whole cell extracts were prepared and the GST-fusion proteins isolated using glutathione resin. Bound fractions were analysed by Western blot analysis with an anti-HA antibody. Expression of each protein was confirmed with anti-GST and anti-HA antibodies. (B) Analysis of the interaction between T and Oct4 and Oct6. 293T cells were transfected with GST-mT together with HA-mMixl1, HA-mOct4 or HA-mOct6. Whole cell extracts were prepared and the GST-fusion proteins were isolated using glutathione resin. Bound fractions were analysed by Western blot analysis with an anti-HA antibody. Expression of each protein was confirmed with anti-GST and anti-HA antibodies. (TIF) [file pone.0028394.s004.tif]

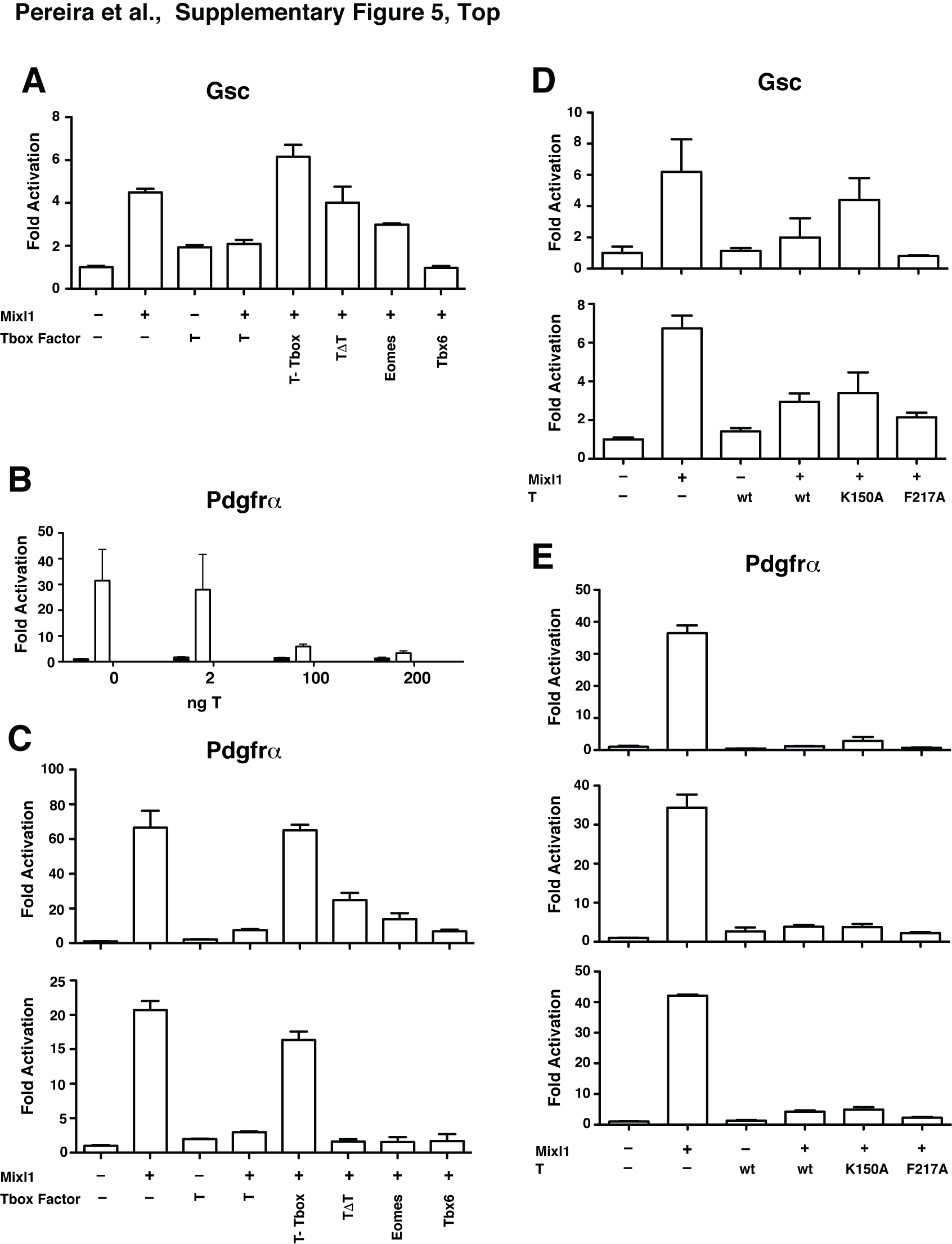

Supplement: Figure S5 — T and related Tbx factors reduce Mixl1 induction of the Gsc and Pdgfrα promoters. (A) An additional replicate of the luciferase reporter assays showing the effect of T domains and related Tbx members on the transactivation activity of Mixl1. Luciferase reporter analysis was performed on the Gsc promoter with 25 ng pMT2-HA-Mixl1 and 200 ng of pMT2-HA-T, T-Tbox, T ΔTbox (TΔT), Eomes or Tbx6. Results from an independent experiment are shown. Error bars show the S.E.M., n = 3. (B) Luciferase reporter analysis was performed on the Pdgfrα promoter with 25 ng pMT2-HA-Mixl1 and increasing amounts of pMT2-HA-T. Results from an independent experiment are shown. Error bars show the S.E.M., n = 3. (C) Luciferase reporter analyses were performed on the Pdgfrα promoter as outlined in (B) with 25 ng pMT2-HA-Mixl1 and 200 ng of pMT2-HA-T, T-Tbox, T ΔTbox (TΔT), Eomes or Tbx6. Results from two independent experiments are shown. Error bars show the S.E.M., n = 3. (D) Additional replicates of the luciferase assay showing the effect of T mutants on the transactivation activity of Mixl1. Reporter analyses were performed on the Gsc promoter with 50 ng pMT2-HA-Mixl1 and 200 ng of pMT2-HA-Brachyury or pMT2-HA-Brachyury T-box mutants K150A and F217A. Results from two independent experiments are shown. Error bars show the S.E.M., n = 3. (E) Luciferase reporter analyses were performed on the Pdgfrα promoter as outlined in (D). Results from three independent experiments are shown. Error bars show the S.E.M., n = 3. (TIF) [file pone.0028394.s005.tif]
